# Widespread age-related differences in the human brain microstructure revealed by quantitative magnetic resonance imaging^☆^

**DOI:** 10.1016/j.neurobiolaging.2014.02.008

**Published:** 2014-08

**Authors:** Martina F. Callaghan, Patrick Freund, Bogdan Draganski, Elaine Anderson, Marinella Cappelletti, Rumana Chowdhury, Joern Diedrichsen, Thomas H.B. FitzGerald, Peter Smittenaar, Gunther Helms, Antoine Lutti, Nikolaus Weiskopf

**Affiliations:** a Wellcome Trust Centre for Neuroimaging, UCL Institute of Neurology, London, UK; b Spinal Cord Injury Center Balgrist, University Hospital Zurich, Zurich, Switzerland; c Department of Brain Repair and Rehabilitation, UCL Institute of Neurology, London, UK; d Department des Neurosciences Cliniques, LREN, CHUV, Universite de Lausanne, Lausanne, Switzerland; e Institute of Cognitive Neuroscience, University College London, London, UK; f Psychology Department, Goldsmiths College, University of London, London, UK; g MR Research in Neurology and Psychiatry, Goettingen University, Goettingen, Germany

**Keywords:** VBQ, Aging, R1, T1, MT, R2*, T2*, Relaxation, Magnetization transfer, Quantitative, 3T, Water content

## Abstract

A pressing need exists to disentangle age-related changes from pathologic neurodegeneration. This study aims to characterize the spatial pattern and age-related differences of biologically relevant measures in vivo over the course of normal aging. Quantitative multiparameter maps that provide neuroimaging biomarkers for myelination and iron levels, parameters sensitive to aging, were acquired from 138 healthy volunteers (age range: 19–75 years). Whole-brain voxel-wise analysis revealed a global pattern of age-related degeneration. Significant demyelination occurred principally in the white matter. The observed age-related differences in myelination were anatomically specific. In line with invasive histologic reports, higher age-related differences were seen in the genu of the corpus callosum than the splenium. Iron levels were significantly increased in the basal ganglia, red nucleus, and extensive cortical regions but decreased along the superior occipitofrontal fascicle and optic radiation. This whole-brain pattern of age-associated microstructural differences in the asymptomatic population provides insight into the neurobiology of aging. The results help build a quantitative baseline from which to examine and draw a dividing line between healthy aging and pathologic neurodegeneration.

## 1 Introduction

Age is the highest risk factor for neurodegenerative disease yet it remains unclear what triggers normal aging processes to diverge into neurodegeneration. In older adults, brain pathology can be present with no apparent cognitive impairment (Fotuhi et al., 2009; Zecca et al., 2004). Macrostructural tissue loss has proved a sensitive marker for neurodegeneration despite having poor pathologic specificity (Barkhof et al., 2009; Benedict and Zivadinov, 2011; Frisoni et al., 2010; McDonald et al., 2009; Scahill et al., 2002). Markers of microstructural changes accompanying atrophy are required to increase sensitivity and specificity (Barkhof et al., 2009; Benedict and Zivadinov, 2011; Frisoni et al., 2010; Noseworthy, 1999; Scahill and Fox, 2007). Our aging population presents a pressing need to disentangle age-related changes from pathologic neurodegeneration. This motivated our study in which we examine normal age-related differences and population variance of quantitative magnetic resonance imaging (MRI) parameters that have been shown to reflect underlying differences in the brain microstructure (Dick et al., 2012; Draganski et al., 2011; Freund et al., 2013; Sereno et al., 2013).

Myelin sheaths exhibit degenerative changes with age that reduce conduction velocity (Adinolfi et al., 1991; Aston-Jones et al., 1985) along affected nerve fibers and may explain some of the cognitive decline seen in older adults (Marner et al., 2003; Peters, 2002). The effects of age on myelin are complex because even though some myelin sheaths are seen to degenerate with age, the process of myelin production continues throughout life, though possibly in an uncontrolled or dysfunctional manner (Peters, 2002). Oligodendrocytes are crucial for the production and maintenance of myelin and require iron to sustain their high metabolic rate and facilitate the synthesis of lipids and cholesterol necessary to carry out these functions (Bartzokis, 2011; Connor and Menzies, 1996; Todorich et al., 2009). This makes iron a key co-factor in the production and maintenance of myelin. Iron levels are highly spatially and developmentally heterogenous, increasing rapidly during development with linear increases in later life, even plateauing in some regions (Hallgren and Sourander, 1958). Oligodendrocytes that differentiate later in life produce thinner sheaths that cover a larger number of thinner axons that are more susceptible to functional impairment and destruction (Bartzokis, 2004; Kemper, 1994; Marner et al., 2003; Terao et al., 1994). Over the course of aging, iron accumulates in brain regions that are susceptible to neurodegenerative diseases (Connor et al., 1990; Dexter et al., 1991; Jellinger and Paulus, 1990; Zecca et al., 2004) though it is not wholly clear whether this accumulation is a cause or an effect of degeneration.

Quantitative MRI can circumvent some of the drawbacks of histologic analysis by producing neuroimaging markers for biologically relevant quantities noninvasively. Recent technical developments have enabled in vivo mapping to be performed with high resolution and whole brain coverage (Deoni et al., 2005; Helms and Dechent, 2009; Helms et al., 2008a, 2008b). Macromolecular protons, such as those found in myelin, can be selectively saturated using off-resonance radiofrequency (RF) pulses leading to attenuation of the magnetic resonance (MR) water signal by magnetization transfer (MT) (Wolff and Balaban, 1989). Voxels with a higher macromolecular content will show a greater percentage loss of water magnetization as a consequence of a given pre-pulse (MT saturation). Magnetization transfer measures have been shown to correlate with histologically measured myelin content (Schmierer et al., 2004, 2007), whereas quantitative relaxation rate measurements correlate with iron content (Daugherty and Raz, 2012; House et al., 2007; Langkammer et al., 2010; Rodrigue et al., 2013; Vymazal et al., 1996).

Gaining insight into the multifaceted and inter-dependent biological processes that underlie both aging and neurodegeneration is a complex problem. Here, we use quantitative multiparameter mapping (MPM), which is ideally suited to probe the multiple factors of aging. MPM quantifies the longitudinal relaxation rate, R_1_, effective transverse relaxation rate, R_2_*, percent saturation because of MT and effective proton density (PD*). We present a cross-sectional whole brain voxel based quantification (VBQ) analysis of these 4 parameters acquired on a large cohort of healthy volunteers covering a broad age range. We hypothesized that age would correlate with regionally specific reductions in myelin content, changes in iron and water content and ultimately with brain atrophy and that these microstructural changes would be reflected by age-related differences in the MPM data.

## 2 Methods

### 2.1 Participants

Participants were recruited from the local university population and by advertising on the departmental website and in local buildings as well as through word of mouth. Potential participants were screened and excluded if they had any of the following: metallic implants, epilepsy, diabetes, history of seizures, neurologic, medical or psychiatric disorders. Of the final pool of participants, all but 2 (1 male, 1 female) were right-handed. To assess cognitive integrity, all older adult participants (60 years or more) additionally underwent a Mini Mental State Examination and achieved scores of 28 or greater. The final cohort consisted of 138 volunteers, of which 49 were men. The group ranged in age from 19 to 75 years with a mean age of 46.6 years and a standard deviation of 21 years. Informed written consent was obtained before scanning.

### 2.2 Data acquisition

Participants were examined on two 3T whole body MR systems (Magnetom TIM Trio, Siemens Healthcare, Erlangen, Germany, 69 participants per scanner) each equipped with a standard 32 channel head coil for receive and RF body coil for transmission. The data were acquired as part of several cognitive neuroimaging studies at the Wellcome Trust Centre for Neuroimaging with approval from the local ethics committee.

A whole-brain quantitative MPM protocol was used. This consists of 3 spoiled multi-echo 3D fast low angle shot (FLASH) acquisitions with 1 mm isotropic resolution and 2 additional calibration sequences to correct for inhomogeneities in the RF transmit field (Lutti et al., 2010, 2012; Weiskopf et al., 2013). The FLASH volumes were acquired with predominantly proton density (PD), T_1_ or MT weighting, determined by the repetition time, and flip angle (α) (repetition time and flip angle were for the PD- and MT-weighted acquisitions: 23.7 ms/6°; and for the T_1_-weighted acquisition: 18.7 ms/20°). In the case of the MT-weighted acquisition, a Gaussian RF pulse with 4 ms duration and 220° nominal flip angle was applied 2 kHz off-resonance before nonselective excitation. Gradient echoes were acquired with alternating readout gradient polarity at 6 equidistant echo times between 2.2 ms and 14.7 ms. Two additional echoes were acquired for the PD-weighted acquisition at 17.2 ms and 19.7 ms. A high readout bandwidth of 425 Hz/pixel was used to reduce off-resonance artefacts (Helms and Dechent, 2009). To speed up data acquisition, parallel imaging with a speed up factor of 2 was used in the phase-encoded direction (anterior-posterior) using the generalized auto-calibrating partially parallel acquisition algorithm. A partial Fourier acquisition (6/8 sampling factor) was used in the partition direction (left-right). The total scanning time of the MPM protocol was approximately 25 minutes.

**Fig. 1:**
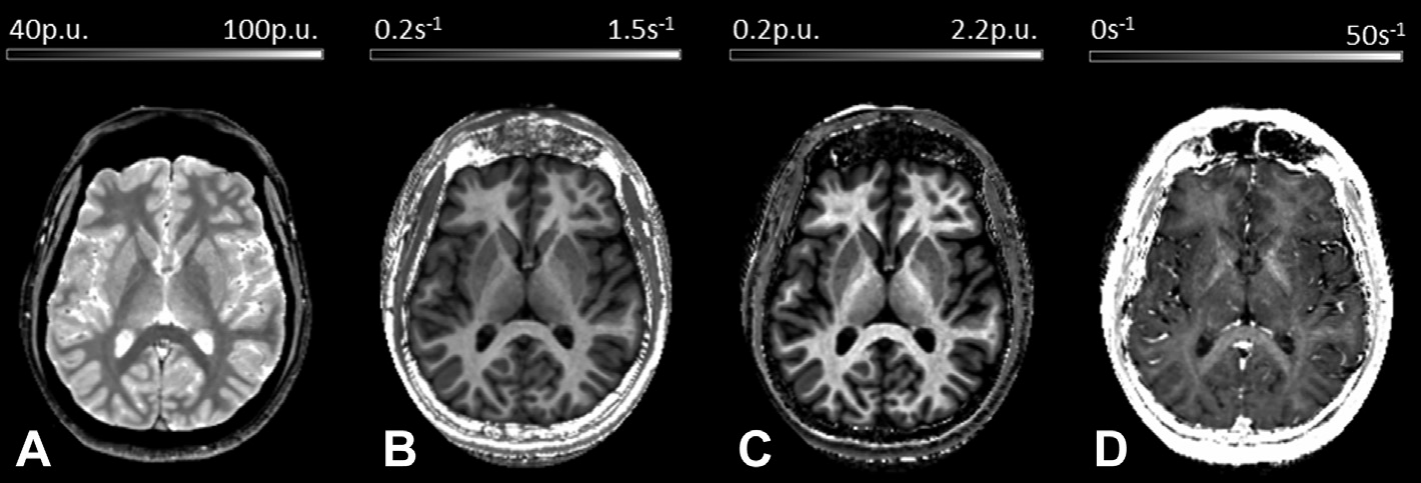
Example maps of an individual volunteer: effective proton density, PD* (A); longitudinal relaxation rate, R_1_ (B); magnetization transfer, MT (C), and transverse relaxation rate, R_2_* (D).

To obtain quantitative maps, the data were processed in the Statistical Parametric Mapping SPM8 framework (Wellcome Trust Centre for Neuroimaging, London) using bespoke MATLAB tools (The Mathworks Inc, Natick, MA, USA). Example maps are shown in Fig. 1. In brief, regression of the log signal from the 8 PD-weighted echoes was used to calculate a map of R_2_*. The set of echoes for each acquired weighting were then averaged to increase the signal-to-noise ratio (Helms and Dechent, 2009). The 3 resulting volumes were used to calculate MT, R1, and PD* maps as described in Helms et al., 2008a, 2008b; Weiskopf et al., 2013. To maximize the accuracy of the R1 map, inhomogeneity in the flip angle was corrected by mapping the B_1_^+^ transmit field according to the procedure detailed in Lutti et al. (2012) and the intrinsically imperfect spoiling characteristics were corrected using the approach described by Preibisch and Deichmann (2009).

The MT map is semi-quantitative depicting the percentage loss of magnetization resulting from the MT pre-pulse used. This differs from the commonly used MT ratio (percentage reduction in steady state signal) by explicitly accounting for spatially varying T_1_ relaxation times and flip angles (Helms et al., 2008b). This results in higher and more robust contrast in the brain than the MT ratio (Helms et al., 2010). PD* maps were estimated from signal amplitude maps by adjusting for receive sensitivity differences using a post-processing method similar to UNICORT (Weiskopf et al., 2011, 2013). To make these maps comparable across participants, they were scaled to ensure that the mean white matter PD* for each subject agreed with the published level of 69% (Tofts, 2003, chapter 4). This quantity is referred to as PD* because there was no correction for R_2_* signal decay. This map was calculated from the averaged multi-echo FLASH data, which has an effective echo time of 8.45 ms.

### 2.3 Age-related differences in brain microstructure

Differences in MR parameters and microstructure were assessed using whole brain VBQ. Differences in local gray matter volume were assessed with voxel-based morphometry (VBM). For voxel-based analysis within the brain, the MT maps were segmented into gray and white matter probability maps using the unified segmentation approach (Ashburner and Friston, 2005). Inter-subject registration of the tissue classes was performed using Dartel, a nonlinear diffeomorphic algorithm (Ashburner, 2007), as implemented in SPM8. This algorithm estimated the deformations that best align the tissue probability maps by iteratively registering them with their average. The resulting Dartel template and deformations were used to normalize the tissue probability maps to the stereotactic space defined by the Montreal Neurological Institute (MNI) template.

For VBM analysis, the normalization procedure included scaling the gray matter tissue probability maps by the Jacobian determinants of the deformation field and smoothing with an isotropic Gaussian smoothing kernel of 6 mm full width at half maximum (FWHM). For VBQ analysis, a different normalization procedure was used on the multiparameter maps to preserve the correct quantitative values. The maps were normalized using the subject-specific deformation fields but without modulating by the Jacobian determinants. Instead a combined probability weighting and Gaussian smoothing procedure described in Draganski et al. (2011) was used with a 3 mm FWHM isotropic smoothing kernel. This method produces tissue-specific parameter maps in MNI space while optimally preserving the quantitative parameter values within each tissue class (reducing effects of residual misregistration and partial volume effects).

### 2.4 SPM analysis in the brain

Statistical analyses were carried out using a multiple linear regression model embedded in the general linear model framework of SPM8. A total of 4 regressors were included in the model. These described age, gender, total intracranial volume, and scanner. One-tailed *t* tests were used to test the hypotheses that MT and R_1_ decrease with age in line with demyelination. A 2-tailed *t* test was used to look for age-related differences in PD* and R_2_* with age. An F-test was used to check for systematic differences in MPMs acquired on the 2 different scanners. In all cases statistical thresholds were applied at *p* < 0.05 after family-wise error correction for multiple comparisons using Gaussian random field theory as implemented in SPM. These tests were carried out voxel-wise across the gray matter (GM) and white matter (WM) sub-space separately using explicit masks defining GM and WM voxels. The masks were generated as follows: smoothed (FWHM of 3 mm isotropic), Jacobian-modulated tissue probability maps in MNI space were averaged across all subjects for each tissue class (GM, WM, and cerebrospinal fluid). Masks were generated by assigning voxels to the tissue class for which the probability was maximal. Voxels for which neither the GM nor the WM probability exceeded 20% were excluded from the analysis. This approach was used to ensure that each voxel was analyzed in only one subspace and that non-brain tissue was excluded.

### 2.5 Labeled analysis in the brain

For comparison with previously published data and to standardize the analysis, the automated anatomical labeling (AAL) atlas (Tzourio-Mazoyer et al., 2002) was used to define anatomic regions. The substantia nigra and red nucleus, which are not present in the AAL atlas, were labeled using the Brodmann atlas supplied with the WFU toolbox for SPM (Maldjian et al., 2003). The AAL labels were also used to extract reference MPM data from GM voxels that showed significant age-related differences (see Supplementary Data).

### 2.6 Age-related differences in the spinal cord

Age-related effects were also examined by investigating differences in quantitative MR parameters within the spinal cord (Freund et al., 2013) and in spinal cord cross-sectional area, which is well established in degenerative disease and trauma (Freund et al., 2011). MPM data were extracted as follows: the central sagittal slice from the averaged T1-weighted FLASH volume was used to manually define the angulation of the spinal cord with respect to the horizontal axis of this view, and to define the cervical vertebra C2 as an anatomic landmark (performed by an expert in spinal cord imaging, PF). The center of C2 was defined on the axial slice containing the anatomic landmark voxel. The boundary of the cord was determined via the nearest-neighbor region growing. The stopping criterion was a 25% drop in signal intensity, which was taken to signify that the WM-cerebrospinal fluid boundary had been reached. The center of the resulting region of interest (ROI) was used to initialize region growing in the adjacent superior slice. A total of 15 slices were included in the analysis giving a total caudal-rostral coverage of 1.5 cm. Spinal cord area was defined as the mean of the area across slices after multiplying by the cosine of the through-plane angulation to account for variation in subject positioning. Mean MT and R_2_* values were extracted from segments coincident with the fibers of the spinal cord tract (bilaterally spanning 45^°^ posteriorly about the midpoint of the cord). R_1_ and PD* were not analyzed because both maps required the correction of RF transmit field inhomogeneities, the mapping of which did not extend to the cord. Linear regression was used to examine the dependency of spinal cord MPM data on age. One-tailed *t* tests were used to test our hypotheses that the spinal cord area decreases with age, is larger in men than in women, that spinal cord MT decreased with age and that spinal cord R_2_* increased with age. The threshold for statistical significance was set at *p* < 0.05.

## 3 Results

**Fig. 2:**
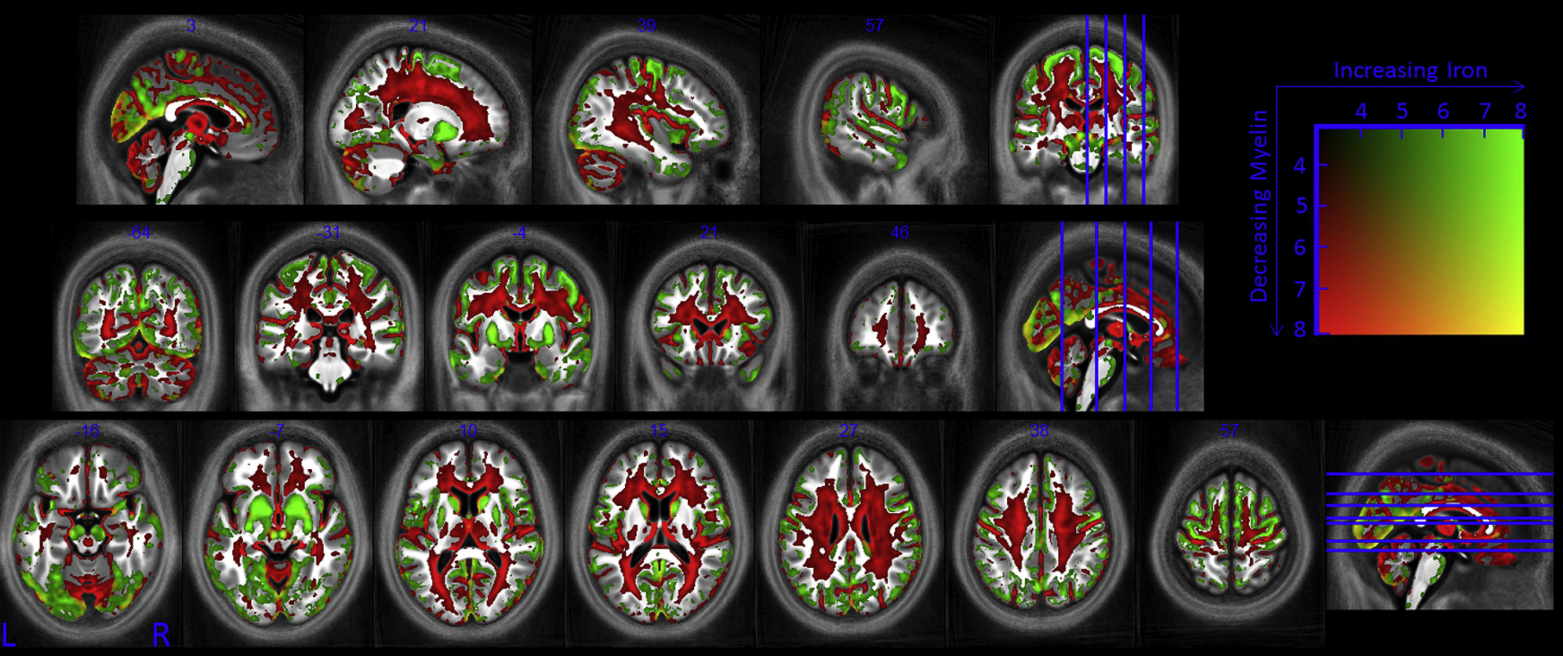
Whole brain pattern of aging. Myelin reductions are estimated from R_1_ and MT decreases while iron increases are estimated from increased R_2_*. This figure is thresholded at the *p* < 0.001 uncorrected level for display purposes only. The *t* score for the combined effects is indicated by the color square. Abbreviations: MT, magnetization transfer; R_1_, longitudinal relaxation rate; R_2_*, transverse relaxation rate.

A whole-brain pattern of aging was identified (Fig. 2) in which all 116 cortical areas defined in the AAL atlas contained voxels in which at least one quantitative parameter showed significant age-related differences (see Supplementary Information).

### 3.1 Voxel-based morphometry

**Fig. 3:**
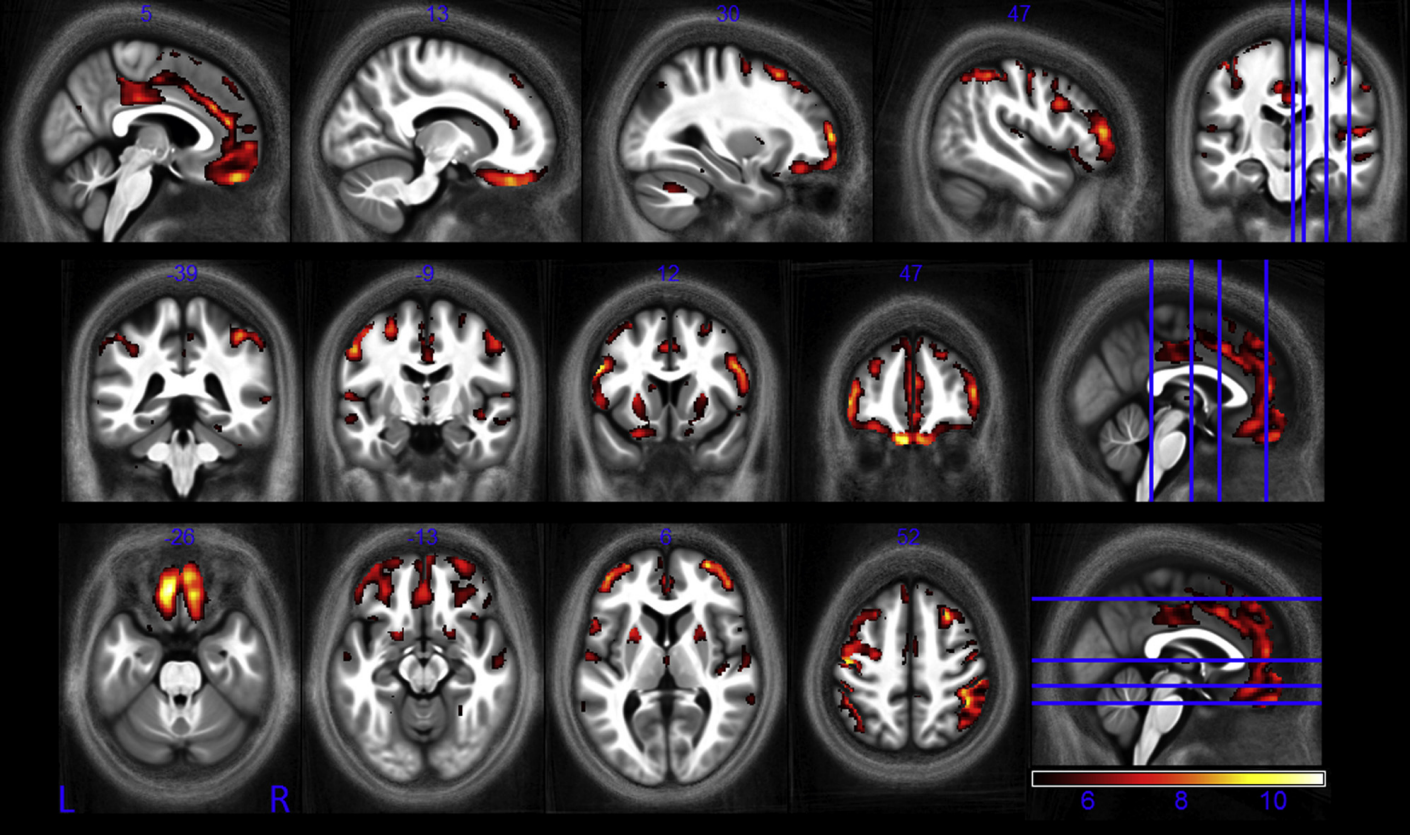
Statistical parameter maps identify regions in which GM atrophy occurs with age at the *p* < 0.05 FWE corrected level. The statistical parametric maps were superimposed on the mean MT map for the cohort in MNI space. Abbreviations: FWE, family-wise error; GM, gray matter; MT, magnetization transfer.

We identified significant age-related GM volume reductions primarily in frontal regions, distributed throughout the cortex and within the putamen (Fig. 3).

### 3.2 Effective transverse relaxation rate

**Fig. 4:**
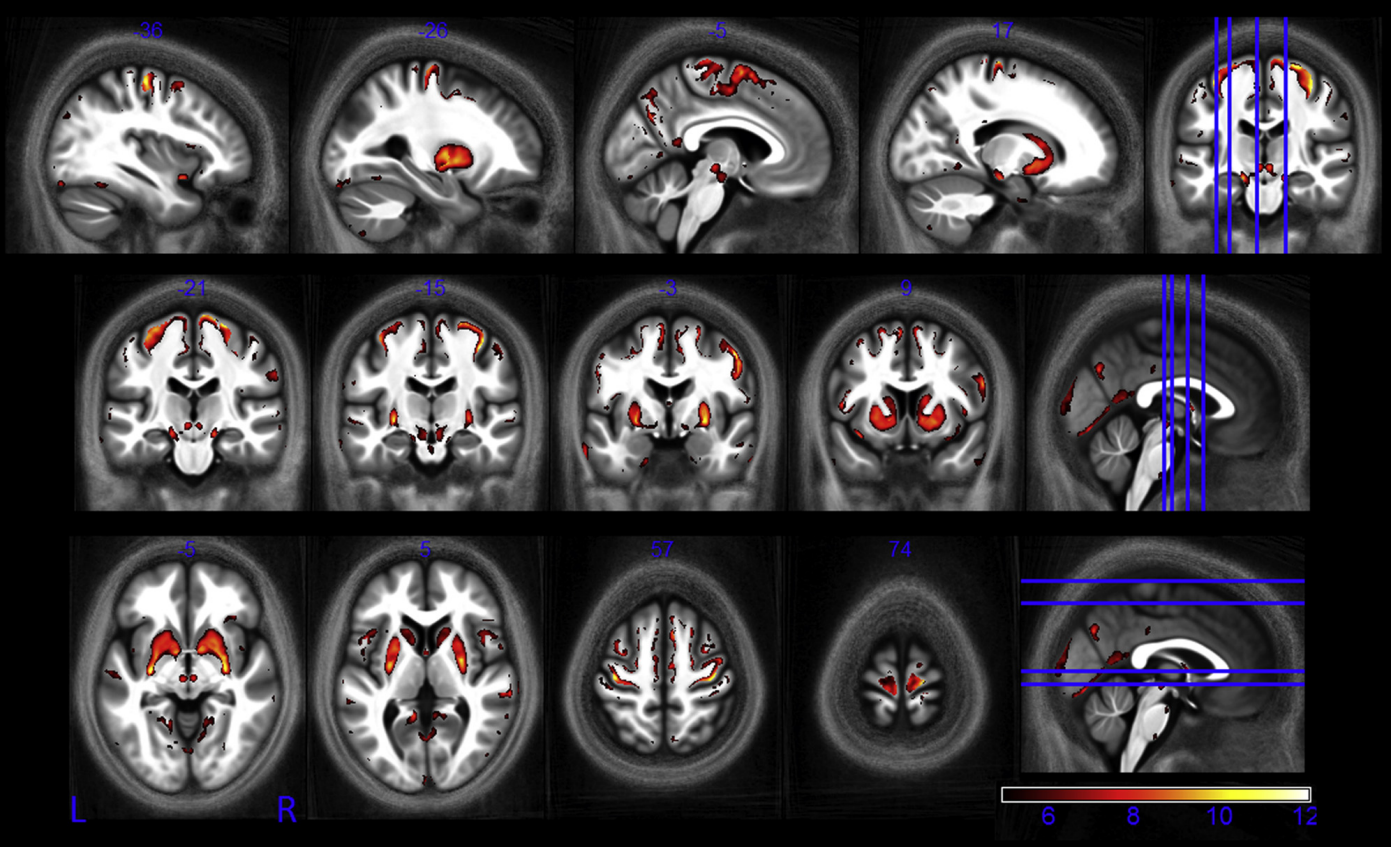
Statistical parametric maps of regions in which R2* significantly increased with age at the *p* < 0.05 FWE corrected level. The SPMs (of both the GM and WM analyses) are superimposed on the mean MT map for the cohort in MNI space. The color bar indicates the *t* score. Abbreviations: FWE, family-wise error; GM, gray matter; MT, magnetization transfer; WM, white matter.

**Fig. 5:**
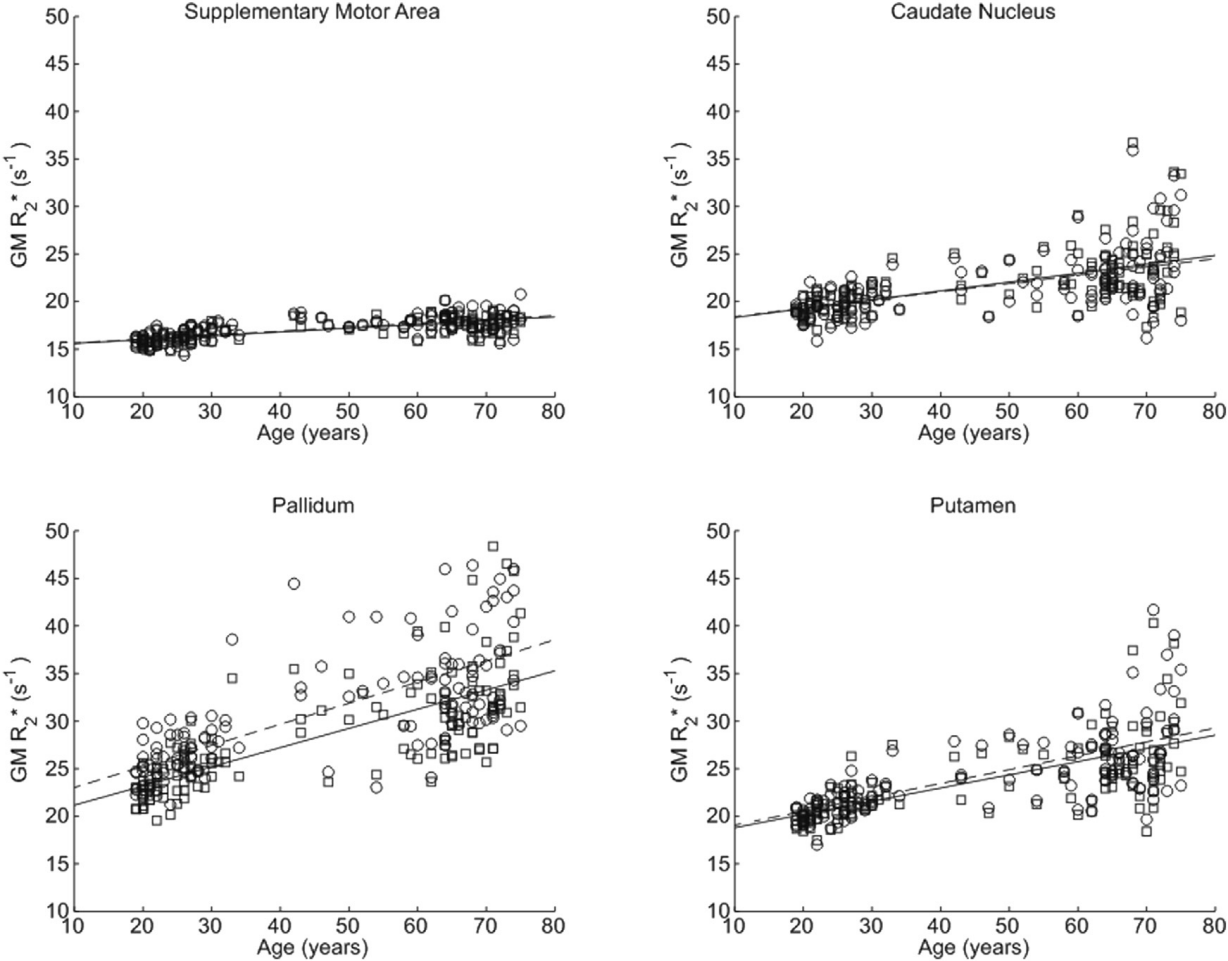
Significant increase in apparent transverse relaxation rate (R2*) in gray matter masked regions of the supplementary motor cortex, caudate nucleus, pallidum, and putamen as a function of age. Similar patterns were observed in the left (circles) and right (squares) hemisphere. The lines (left dashed, right solid) depict the linear model fit. These data are shown for illustration purposes only and were not used for any additional analyses.

The R_2_* values measured in this study showed good agreement with published values; though typically our values were somewhat lower (Table 1). We found significant positive correlations between R_2_* and age in the putamen, the pallidum, the caudate nucleus, the red nucleus as well as in extensive cortical regions, particularly in the motor cortex (Fig. 4). Most age-related differences were seen bilaterally. Exemplar data extracted from the normalized R_2_* maps for the supplementary motor cortex, caudate nucleus, pallidum, and putamen along with the respective linear age dependence derived from the SPM analysis are plotted in Fig. 5. Across all structures the range of significant R_2_* increase was from 0.03 to 0.22 s^−1^ per year.

**Fig. 6:**
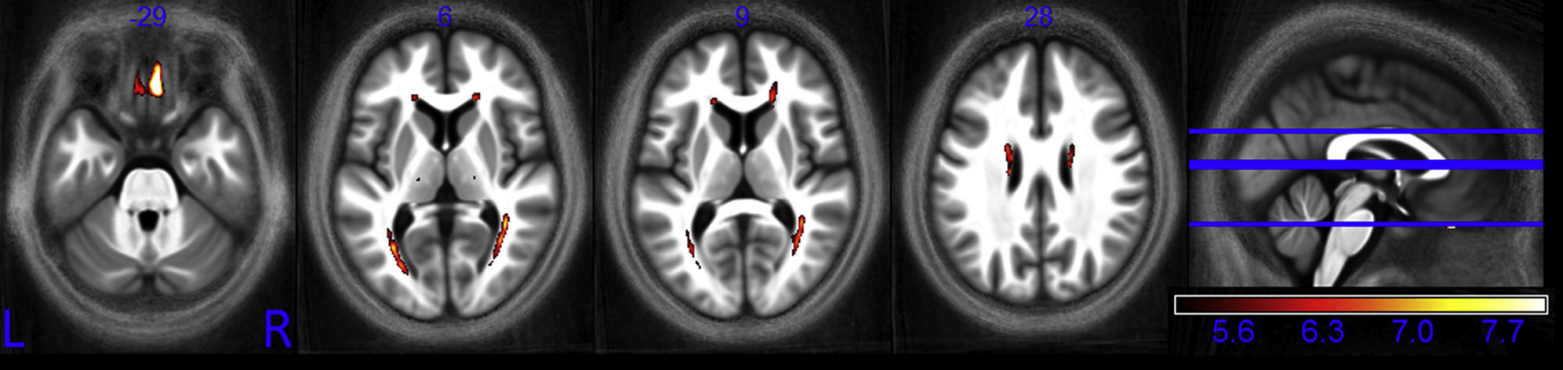
Statistical parametric maps of regions in which R_2_* significantly decreased with age at the *p* < 0.05 FWE corrected level. The SPMs (of both the GM and WM analyses) are superimposed on the mean MT map for the cohort in MNI space. The color bar indicates the *t* score. Abbreviations: FWE, family-wise error; GM, gray matter; MT, magnetization transfer; WM, white matter.

Less widespread R_2_* decreases were also seen (Fig. 6). These occurred within the medial part of the ventral surface of the frontal lobe, along the superior occipitofrontal fascicle, the optic radiation, and at a small number of focal locations within the corpus callosum and the corticospinal tract.

### 3.3 MT saturation

**Fig. 7:**
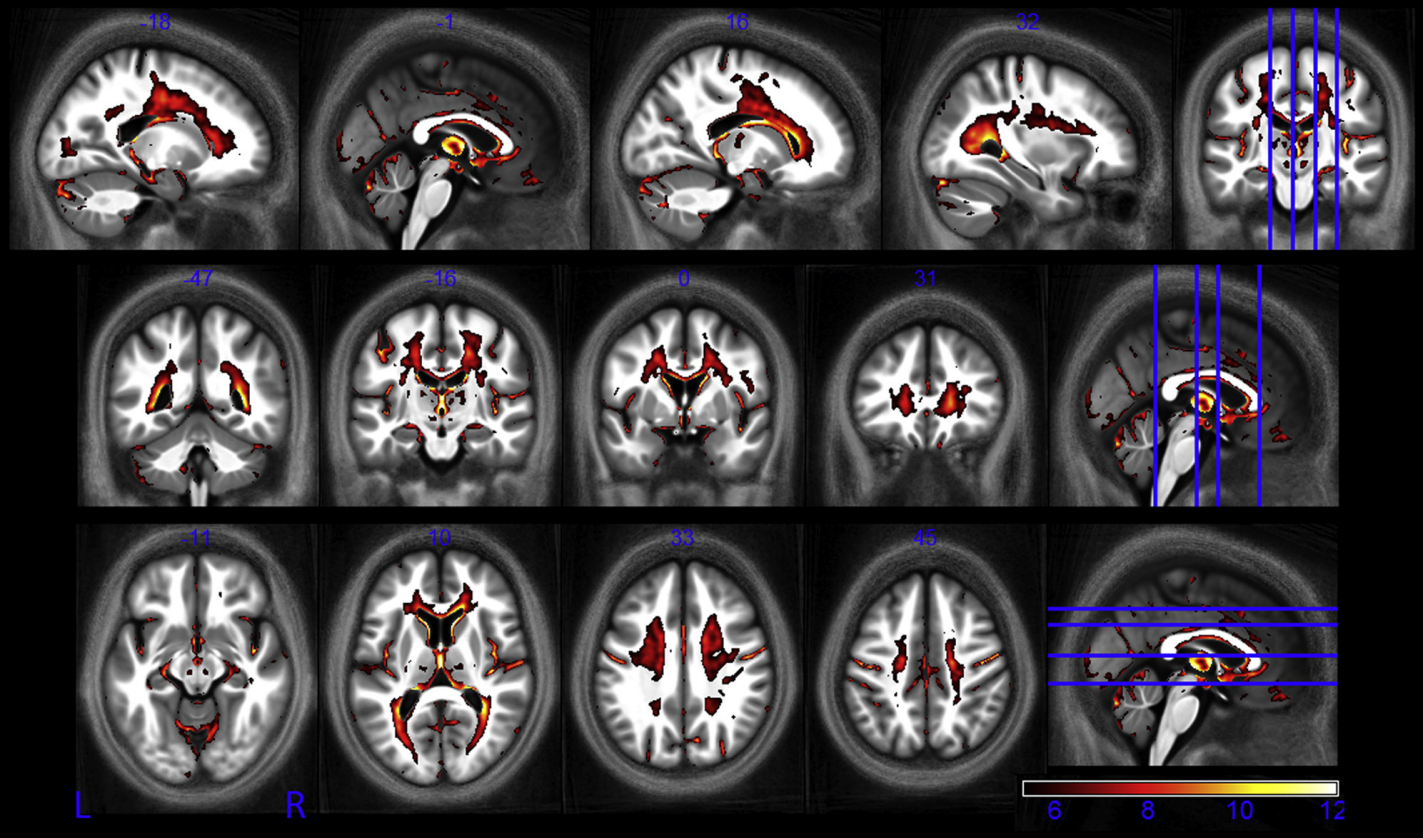
Statistical parametric maps of regions in which MT significantly decreased with age at the *p* < 0.05 FWE corrected level. The SPMs are superimposed on the mean MT map for the cohort in MNI space. The color bar indicates the *t* score. Abbreviations: FWE, family-wise error; MT, magnetization transfer.

**Fig. 8:**
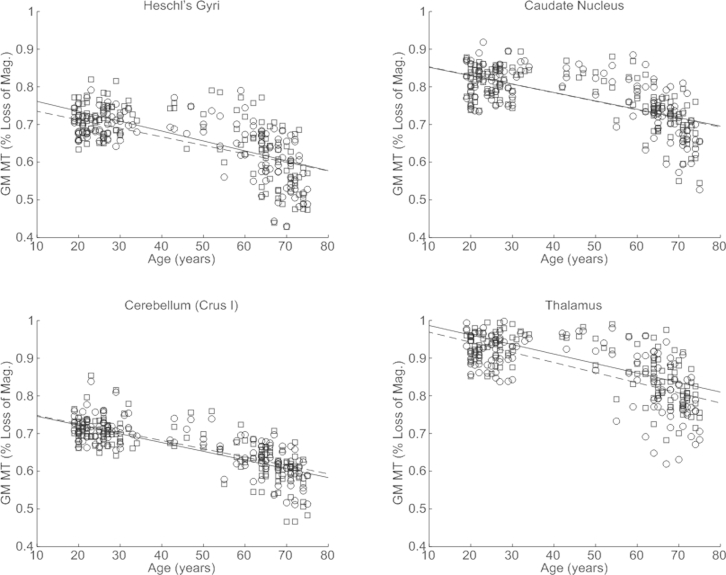
Significant decrease in magnetization transfer (MT) in Heschl gyri, the caudate nucleus, cerebellum (crus I), and thalamus as a function of age. Similar patterns were observed in the left (circles) and right (squares) hemisphere. The lines (left dashed, right solid) depict the mean behavior modeled by the linear fit. There appear to be nonlinear effects and rapid decline after 60 years in all regions. These data are shown for illustration purposes only and were not used for any additional analyses.

Extensive significant negative correlations between MT and age were identified within WM, particularly in frontal and parietal regions (Fig. 7). Bilateral MT reduction occurred within the optic radiation, the genu and body of the corpus callosum, and portions of the corticospinal tract. Significant negative correlations were also seen in cortical regions and in the thalamus both at focal central locations and along the medial and posterior periphery. Data extracted from the normalized MT maps are given for the Heschl gyri, the caudate nucleus, cerebellum (crus I), and thalamus along with the respective linear dependence derived from the SPM analysis (Fig. 8). The significant age-dependent decrease in MT ranged from 0.0013% to 0.0031% per year. Post hoc comparison showed that the rate of MT reduction over the course of aging was significantly higher in the genu than the splenium at 0.0019% and 0.0005% per year, respectively (*p* = 0.008).

### 3.4 Longitudinal relaxation rate

**Fig. 9:**
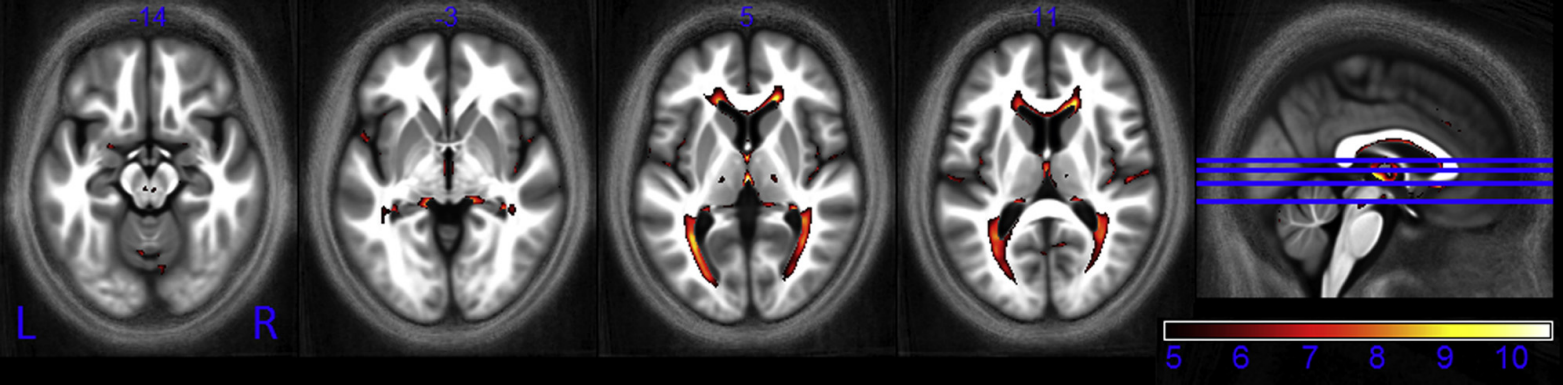
Statistical parametric maps of regions in which R_1_ significantly decreased with age at the *p* < 0.05 FWE corrected level. The SPMs were superimposed on the mean MT map for the cohort in MNI space. The color bar indicates the *t* score. Abbreviations: FWE, family-wise error; MT, magnetization transfer.

Negative correlations between R_1_ and age were primarily identified bilaterally along the optic radiation and in the genu of the corpus callosum (Fig. 9). The age-correlated decrease in R_1_ ranged from 0.0007 to 0.0016 s^−1^ per year.

### 3.5 Effective proton density

**Fig. 10:**
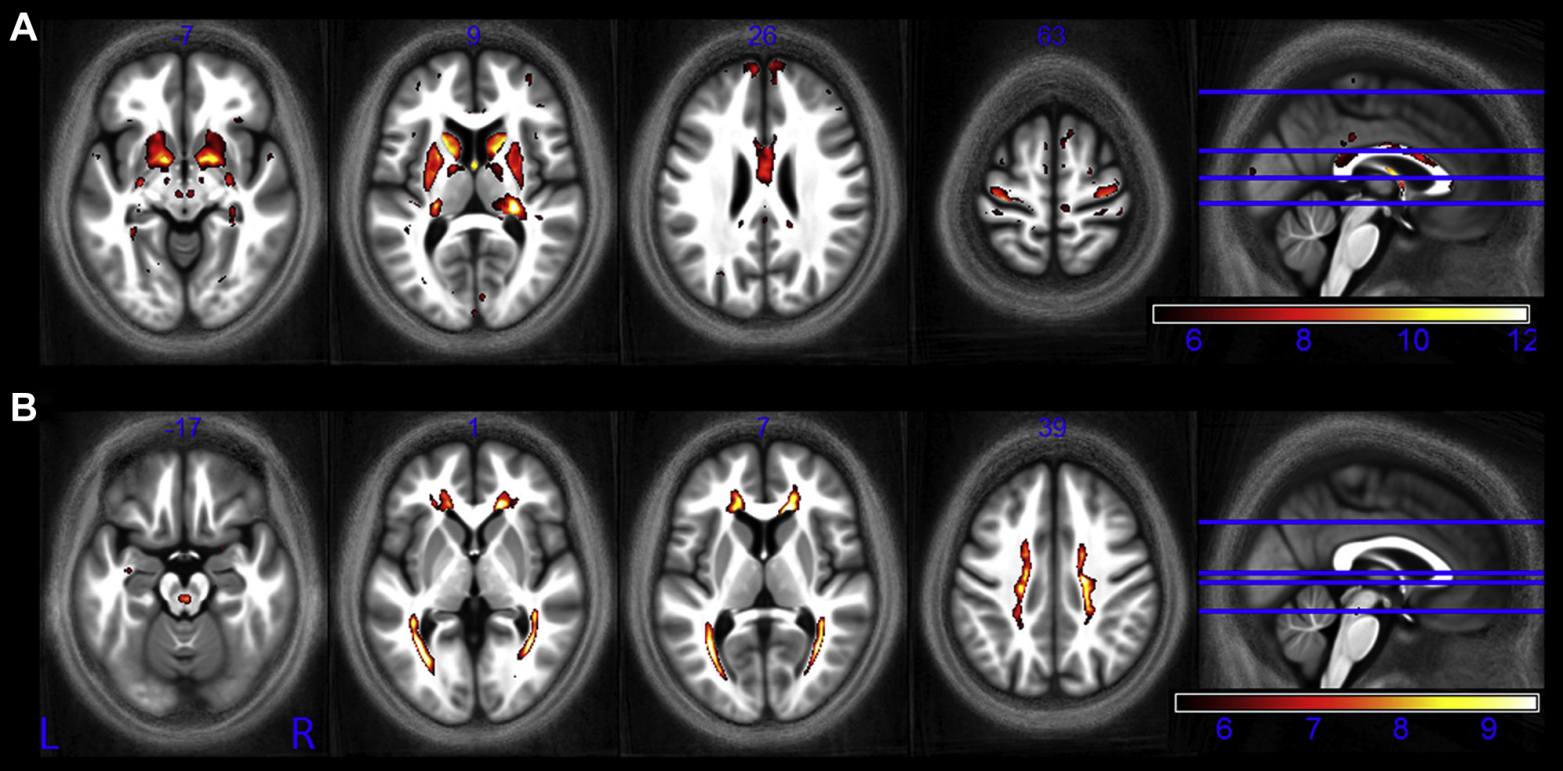
Statistical parametric maps identify regions in which PD* significantly (A) decreased with age and (B) increased with age at the *p* < 0.05 FWE corrected level. The SPMs were superimposed on the mean MT map for the cohort in MNI space. The color bar indicates the *t* score for the decrease with age in A and the increase with age in B. Abbreviations: FWE, family-wise error; MT, magnetization transfer.

We identified negative correlations between effective proton density and age in the putamen, pallidum, caudate nucleus, and the red nucleus (Fig. 10A). We also identified positive correlations between effective proton density and age in the optic radiation and superior regions of white matter (Fig. 10B).

### 3.6 Spinal cord

The measured spinal cord area was 79.2 ± 7.2 mm^2^ (mean ± standard deviation). The cord area was significantly larger in men (80.7 ± 6.1 mm^2^ vs. 76.6 ± 6.9 mm^2^, *p* < 0.01, *t* score = 3.28) but did not significantly decrease with age. Linear regression showed a significant increase in spinal cord R_2_* with age (*p* < 0.05, linear coefficient = 0.031s^−1^ per year, *t* score = 2.91) but not MT.

### 3.7 Scanner differences

A small number of voxels showed significant differences between the 2 scanners used for data acquisition. In all cases, these were randomly distributed across the brain and maximally encompassed less than 0.07% of the analyzed volume. In the R_2_* data, 2 clusters correlated with scanner (3 voxels in GM, F = 31.00 and 2 voxels in WM, F = 27.19). The MT data contained 29 scanner-related cluster (383 voxels in total, peak F = 39.66) in the WM but no voxels were identified in GM. Nine clusters (31 voxels in total, peak F = 35.23) were identified in the GM sub-class of the PD* maps but none in WM. Finally, 2 clusters were identified in the R_1_ maps in GM (121 voxels, F = 58.05 and 5 voxels, F = 32.40) but none in WM and no voxels were identified in the VBM analysis.

## 4 Discussion

The MPM approach demonstrates widespread age-related differences in the microstructure of the human brain. The observed differences in the quantitative MR parameters are in line with ex vivo histologic reports and have high specificity for tissue properties such as macromolecular, iron, and water content. The results will inform future studies about age-related differences in R_2_*, R_1_, PD*, and MT. This quantitative multiparameter mapping approach offers a promising opportunity for concurrently investigating a number of tissue properties and validating the MRI-based measures against their histologic counterparts.

Decreases in MT reflect a loss of macromolecular content, typically myelin. Later-myelinating regions such as the temporal and frontal lobes appear more susceptible to myelin breakdown than earlier-myelinating regions such as the motor and sensory systems (Flechsig, 1901; Kemper, 1994). The pattern of decreased MT we have observed is consistent with this, showing high levels of demyelination in frontal WM, a finding that is in keeping with an anterior-posterior gradient of age-related differences in white matter (Bartzokis et al., 2012; Head, 2004; Raz, 2000). Under the hypothesis of later differentiating oligodendrocytes being more vulnerable, it is expected that the genu of the corpus callosum connecting the prefrontal lobes should be at greater risk of demyelination than the splenium (Bartzokis et al., 2012; Head, 2004), connecting the occipital lobes because the latter is myelinated earlier in life. Our data is also consistent with this. Significant demyelination manifesting as reductions in both MT (Fig. 7, axial slice, z = +10) and R_1_ (Fig. 9, axial slice, z = +11) can be seen in the genu but not in the splenium where the rate of MT reduction with age was significantly lower.

The interpretation of level and change in iron content is complex. Under normal conditions, oligodendrocytes strongly stain for iron, ferritin, and transferrin in the human brain (Connor et al., 1990; Gerber and Connor, 1989; LeVine and Macklin, 1990; Morris et al., 1992). Therefore, some of the observed age-related differences in R_2_* may reflect age-related underlying differences in oligodendroglia, and perhaps by extension also myelination levels because of the determinative role of oligodendrocytes on myelin status. However, in various neurologic conditions iron increases are associated with demyelination when iron is released by damaged oligodendrocytes (Beard et al., 2009; Zecca et al., 2004); is present in the form of hemosiderin deposition (an iron storage complex with poor bioavailability which is associated with micro-bleeds in AD) (Frisoni et al., 2010); or selectively accumulated in particular structures (Jellinger and Paulus, 1990). By having multispectral measures we can better distinguish these conflicting processes. For example, primary motor regions are early-myelinating and can be expected to maintain myelin levels to a more advanced age (Kemper, 1994). Our data are indicative of significant iron increases in the motor cortex but no differences in myelin content. In this case the increased iron is likely present within the iron-rich oligodendrocytes, which serve to maintain the myelin sheath of the motor area. Positive staining for ferritin and iron have been reported to occur within astrocytes and microglia in the cerebral cortex of aged brains (Connor et al., 1990) and may in part explain the observed increase in R_2_*. Conversely, the pattern of age-dependent demyelination (decreased MT) concurrent with increased iron (R_2_*) that could be attributed to iron release from damaged oligodendrocytes was not prevalent in our healthy cohort but was seen only in the red nucleus of the mid-brain. Our data also show that where reductions in R_2_* occurred, they were coincident with reduced MT. This is perhaps indicative of a loss of oligodendrocytes and associated iron, concomitant with demyelination. This pattern was seen primarily in the optic radiation and the superior occipitofrontal fascicle. These structures also showed significant decreases in R_1_, which is dependent on both iron and myelin stores. The age-related differences of all 3 parameters are in line with recent models of myelin describing its effect on local magnetic susceptibility (Wharton and Bowtell, 2012) and previous studies on relaxometry and magnetization transfer observed in myelin (Koenig et al., 1990; Schmierer et al., 2004).

Our hypothesis of significantly increased iron stores in the basal ganglia, in keeping with the seminal histologic study by Hallgren and Sourander (1958), was confirmed. Age-dependent increases in R_2_* have also been reported for the hippocampus (Bartzokis et al., 2007; Rodrigue et al., 2013). We did not see such correlations; however, these studies used ROI analysis as opposed to the whole brain approach adopted here. The correction for multiple comparisons across voxels and spatial smoothing may have reduced the sensitivity of the statistical test in comparison to a targeted ROI analysis.

Thalamic volume loss is associated with cognitive impairment (Benedict and Zivadinov, 2011). We saw significant atrophy occurring with age in our asymptomatic, healthy cohort. This was coincident with significantly decreased MT values, suggesting a concomitant loss of myelination in this structure. Spinal cord atrophy has also been reported as a sensitive marker for disease progression in multiple sclerosis (Barkhof et al., 2009). Our cohort did not show a decrease in spinal cord area suggesting that age-related atrophy was not occurring, though we cannot exclude the possibility of small differences that cannot be observed with this study's statistical power.

Head position in the scanner, specifically translation in y and z and rotation about x, was systematically related to age, our parameter of interest. We believe this was because of postural changes occurring with age leading to systematic positioning effects within the scanner. It is possible that position within the bore could affect the measured R_2_*. For example, orientation dependent effects of myelinated fiber tracts on R_2_* have been described (Bender and Klose, 2010; Wharton and Bowtell, 2013). However, the average change in head orientation was only circa 0.17° per year. Using the model of Bender and Klose, 2010 an upper estimate of the change in R_2_* that could result is 0.008 s^−1^ per year, far lower than the significant age-related differences observed in this cohort, which ranged from 0.03 to 0.22 s^−1^ per year. Moreover, the R_2_* values measured in this study are in good agreement with published values (Gorell et al., 1995; Khalil et al., 2011; Péran et al., 2007; Ordidge et al., 1994; see Table 1). There is only a slight bias toward lower values in our study. We explain this by the higher resolution 3D approach we have used, which reduces the effects of spurious background magnetic field gradients.

There was negligible dependence on which scanner was used for data acquisition, in line with a previous MPM multicenter study demonstrating minimal inter-site variation (Weiskopf et al., 2013). Those voxels that did significantly correlate with scanner choice were sporadic rather than spatially structured. The scanner independence bodes well for multicenter and longitudinal studies, a typical requirement of any study monitoring disease progression or treatment response (Barkhof et al., 2009).

It has been suggested that age-dependent changes follow a nonlinear trajectory (Bartzokis et al., 2012; Fjell et al., 2014; McDonald et al., 2009; Ziegler et al., 2012), whereas here we consider only linear age-related differences. Although our cohort included participants from each decade of the age span studied (19–75 years) the bracket 35 to 55 years was more sparsely sampled, which is likely to have made our analysis particularly sensitive to linear rather than higher order differences. However, the reduction in MT saturation seen in this study is indeed suggestive of accelerated demyelination with advancing age. A sharp decrease can be seen after the age of 60 years (Fig. 8). Early nonlinear age-dependence is also seen in the R_2_* profile in the putamen (Fig. 5) in keeping with the slower iron accumulation reported for this structure (Hallgren and Sourander, 1958). However, the absence of children and adolescents from our cohort means that we cannot adequately sample early nonlinear effects such as the proposed “inverted-U” myelination trajectory (Bartzokis, 2011; Westlye et al., 2010) or exponential iron accumulation (Hallgren and Sourander, 1958). Increased biological variation is seen at more advanced ages. Despite the fact that our participants had no evidence of cognitive impairment, the influence of pre-clinical degeneration on this increased variation cannot be ruled out. We also cannot exclude the possibility of biased sampling. For example, preferential sampling of high functioning older adults (minimum Mini Mental State Examination score of 28), given the fact that our cohort was recruited from participants of other studies within the Wellcome Trust Centre for Neuroimaging. These complex aging effects further highlight the difficulty of making inferences about population variance from small cohort numbers in histologic studies. Longitudinal rather than cross-sectional studies are best placed to elicit insight into the true aging trajectory, which is impossible with invasive histology but feasible using the MPM approach.

The effective proton density (PD*) measure used in this study does not account for multi-exponential signal decay and has residual T_2_* weighting because of its effective 8.45 ms echo time. Although the differential effect between GM and WM is small (∼3%) it is likely that this is why there is considerable overlap between regions, such as the basal ganglia, that show decreased PD* and increased R_2_*.

Some of the identified age-related differences may be because of partial volume effects because of the coincidence of age-dependent atrophy and registration errors. However, these sources of bias were minimized by using the highly nonlinear diffeomorphic inter-subject registration algorithm (Dartel), which has been shown to result in maximally accurate registration (Klein et al., 2009), and by using a 20% threshold on the average probability map to conservatively partition the data into gray and white matter before statistical analysis.

## 5 Conclusions

We present quantitative multiparameter maps that act as sensitive neuroimaging markers of age-related differences in the brain and spinal cord microstructure over the course of normal aging. There is complex interplay between various neurobiological factors such as oligodendrocyte integrity, ferritin levels, myelin production, and maintenance and neurodegeneration. The multispectral maps provide complementary information that allows the underlying, interdependent biological features to be more fully investigated. Myelin and iron content combine in a mixed manner to determine the measured R_1_ and PD* but directly influence MT and R_2_*, respectively, increasing the sensitivity of these latter parameters to age-related differences. MT acts as an anatomically-specific marker for myelin and identified widespread regions in which demyelination occurred with age, including differential demyelination within the corpus callosum that is in keeping with histologic findings. The iron marker R_2_* identified increased iron levels with age, particularly in the cortex and basal ganglia. The MPM parameters combine to give a multispectral whole-brain view of the neurobiology of aging (Fig. 2). Many opportunities arise from understanding these age-related mircrostructural differences including identifying pathologic deviations from the expected course of aging, monitoring disease progression, and response to treatment and stratifying disease sub-types. The VBQ approach used here could be extended to study neurodegenerative disorders. This study provides reference values and an estimate of population variance from a healthy cohort over and above which we should expect to see differences in pathologic conditions if they are to be used as reliable measures to monitor disease status and progression. Given the profound age-related differences identified in this study, future quantitative studies are motivated to include age as a confounding factor to differentiate disease effects within the aging population.

## Disclosure statement

All authors report no actual or potential conflicts of interest.

## Figures and Tables

**Supplementary Fig. 1:**
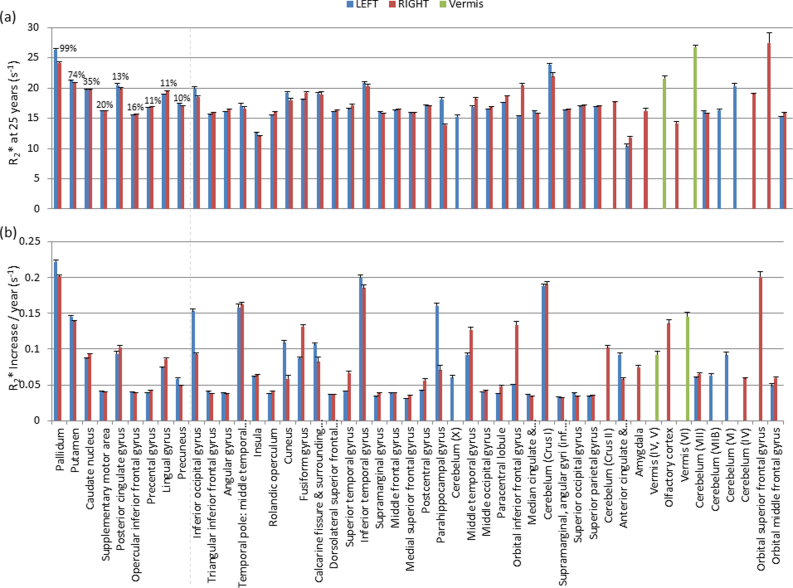
Parameters of the linear fit to R_2_* in gray matter masked areas defined by the AAL atlas that showed significant increase with age. R_2_* at the age of 25 years is shown in (A) and the rate of increase of R_2_* with age is shown in (B). The ROIs are ordered from left to right with decreasing percentage of ROI that significantly correlated with age. The mean (across hemispheres) percentage of significant voxels is noted for those ROIs exceeding 10%. The peak *t* score within the ROIs ranged from 5.4 to 11.2. Abbreviations: AAL, automated anatomical labeling; ROI, region of interest.

**Supplementary Fig. 2:**
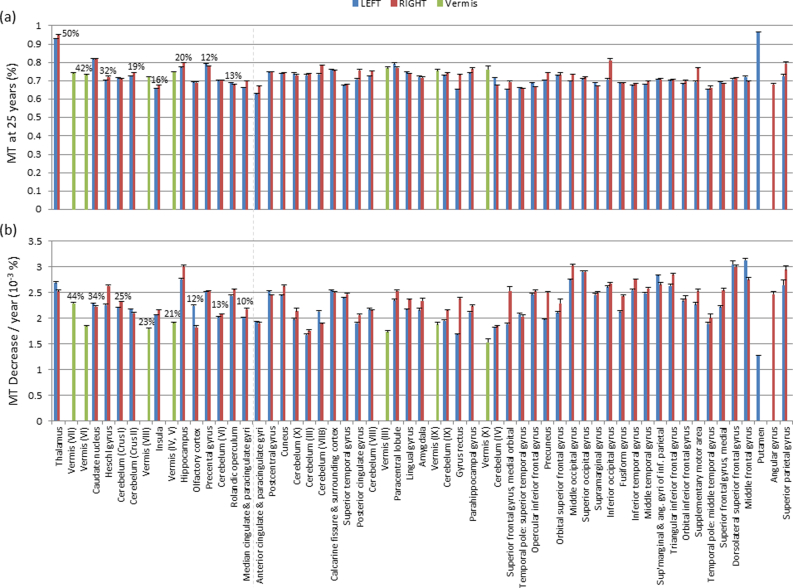
Parameters of the linear fit to MT in gray matter masked areas defined by the AAL atlas that showed significant decrease with age. MT at the age of 25 years is shown in (A) and the rate of decrease of MT with age is shown in (B). The ROIs are ordered from left to right with decreasing percentage of ROI that significantly correlated with age. The mean (across hemispheres) percentage of significant voxels is noted for those ROIs exceeding 10%. The peak *t* score within the ROIs ranged from 5.7 to 13.9. Abbreviations: AAL, automated anatomical labeling; MT, magnetization transfer; ROI, region of interest.

**Supplementary Fig. 3:**
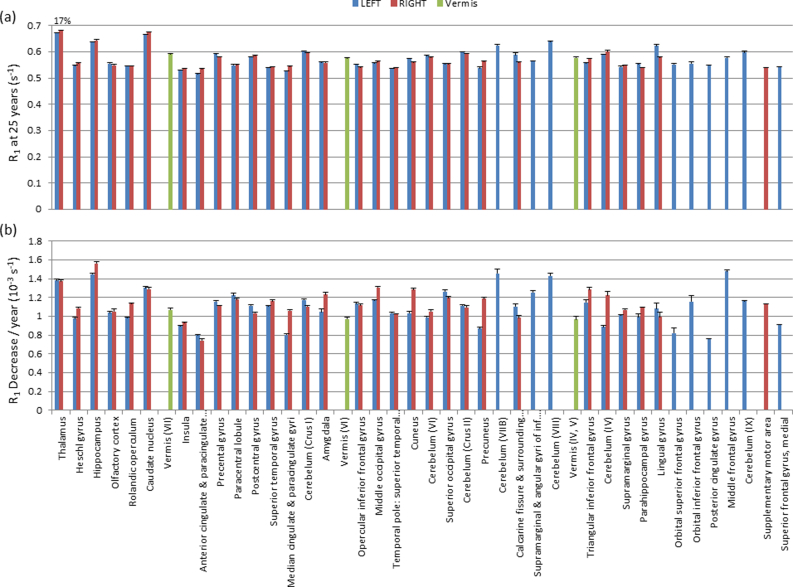
Parameters of the linear fit to R1 in gray matter masked areas defined by the AAL atlas that showed significant decrease with age. R1 at the age of 25 years is shown in (A) and the rate of decrease of R1 with age is shown in (B). The ROIs are ordered from left to right with decreasing percentage of ROI that significantly correlated with age. Across both hemispheres, a mean of 17% of thalamus voxels had a significant negative correlation with age. Less than 6% of consitutent voxels were significantly correlated for the other ROIs. The peak *t* score within the ROIs ranged from 5.4 to 10.9. Abbreviations: AAL, automated anatomical labeling; ROI, region of interest.

**Supplementary Fig. 4:**
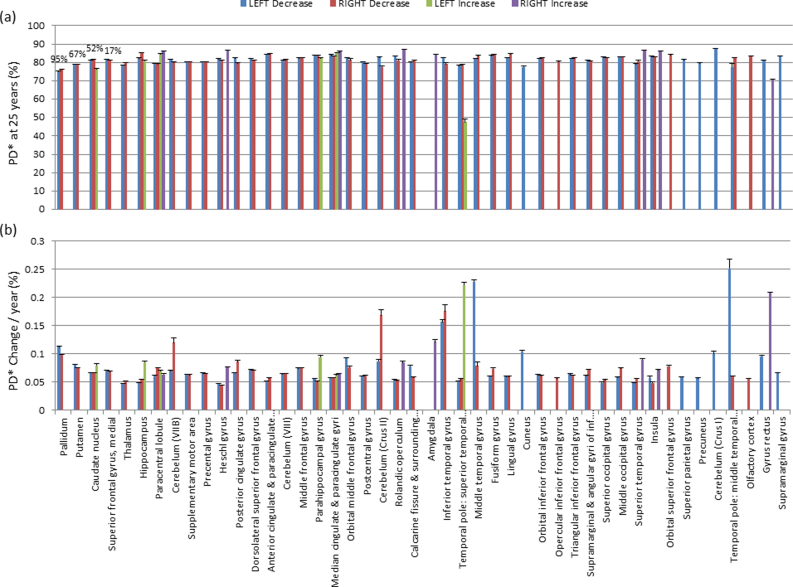
Parameters of the linear fit to PD* in gray matter masked areas defined by the AAL atlas that showed significant change with age. PD* at the age of 25 years is shown in (A) and the rate of increase (green, purple) or decrease (blue, red) of PD* with age is shown in (B). The ROIs are ordered from left to right with decreasing percentage of ROI that significantly correlated with age. The mean percentage (across hemispheres) of the ROIs with more than 10% of constituent voxels showing a negative correlation with age are noted. No positive correlation had more than 1.2% of consituent voxels showing significance. The peak *t*score within the ROIs ranged from 5.5 to 11.8. Abbreviations: AAL, automated anatomical labeling; ROI, region of interest.

**Table 1:** Comparison of published R_2_* values with age-matched data measured via the MPM protocol

Label	Literature value	Age-matched VBQ measure
Globus pallidus^a^	33.0 (30.9–35.2)	28.32 (21.71–49.84)
Left^b^	35.47 (3.3)	26.99 (2.94)
Right^b^	33.99 (3.7)	25.70 (2.65)
Putamen^a^	23.7 (22.2–26.1)	20.74 (17.49–26.90)
Left^b^	25.06 (2.2)	20.65 (1.50)
Right^b^	24.08 (2.2)	19.02 (1.30)
Caudate^a^	21.1 (20.0–22.9)	18.75 (16.06–22.25)
Left^b^	24.78 (2.5)	17.77 (1.10)
Right^b^	24.66 (2.9)	18.72 (1.11)
Thalamus^a^	20.5 (19.9–21.0)	18.50 (16.05–22.22)
Left^b^	20.96 (1.2)	18.07 (1.14)
Right^b^	22.12 (1.4)	18.30 (1.22)
Substantia nigra^c^	37.59 (3.11)	26.70 (4.08)
Left^b^	31.78 (3.7)	24.14 (3.08)
Right^b^	31.47 (2.9)	23.66 (2.89)
Red nucleus		
Left^b^	29.67 (3.3)	17.39 (1.44)
Right^b^	27.39 (3.3)	16.36 (1.53)

VBQ measures are from areas defined by the AAL atlas. The substantia nigra and red nucleus are defined by the Wake Forest University (WFU) atlas. Key: AAL, automated anatomical labeling; MPM, multiparameter mapping; VBQ, voxel-based quantification. Study details (N = number of subjects) are given in the following footnotes:

a Khalil, 2011; N = 35, 36.7 ± 13.7 years, N_VBQ_ = 93.

b Peran, 2007; N = 18, 20–41 years, N_VBQ_ = 58.

c Ordidge, 1994; N = 7, 47–72 years, N_VBQ_ = 63.
